# Toxicity Profiling and *In Vivo* Metabolism of Danshensu-Derived Novel Antihypertensive Candidate 221s (2,9)

**DOI:** 10.3390/toxins17090436

**Published:** 2025-09-01

**Authors:** Yunmei Chen, Kuan Yang, Lili Yu, Rong Wang, Shaojing Liu, Bei Qin

**Affiliations:** 1 Xi’an Key Laboratory for Research and Development of Innovative Multi-Target Antihypertensive Drugs, Xi’an Innovative Antihypertensive Drugs International Science and Technology Cooperation Base, Xi’an Medical University, Xi’an 710021, China; yunmeichen@xiyi.edu.cn (Y.C.); yangkuan@xiyi.edu.cn (K.Y.); yulili@xiyi.edu.cn (L.Y.); wangrong@xiyi.edu.cn (R.W.); liushaojing@xiyi.edu.cn (S.L.); 2 Institute of Drug Research, Xi’an Medical University, Xi’an 710021, China; 3 College of Pharmacy, Xi’an Medical University, Xi’an 710021, China

**Keywords:** 221s (2,9), acute toxicity, long-term toxicity

## Abstract

Compound 221s (2,9) is a novel antihypertensive drug candidate synthesized utilizing danshensu, borneol, and proline by using the strategy of combinatorial molecular chemistry. This study aimed to systematically identify the safety of danshensu-derived compound 221s (2,9) by conducting an acute toxicity test and long-term toxicity study and to elucidate the in vivo metabolic pathways of 221s (2,9) in order to provide critical insights into the observed toxicity. In the acute toxicity study, a single oral dose of 221s (2,9) at 3000 mg/kg in mice produced no clinical signs of toxicity or mortality, indicating an MTD of 3000 mg/kg. In a subsequent 12-week repeated-dose toxicity study in rats, doses of 20, 40, and 80 mg/kg were well tolerated, with no adverse clinical observations or deaths. Notably, organ coefficient analysis revealed transient lung injury, which resolved following a 4-week recovery period. The metabolite identification study indicated that metabolism in rats is predominated by Phase II metabolites, potentially contributing to the low toxicity of 221s (2,9). Further investigation into the impact of the drug metabolic enzyme–transporter interplay on the in vivo disposition of 221s (2,9) is warranted.

## 1. Introduction

Hypertension, a persistent elevation of arterial blood pressure, constitutes a chronic condition that has escalated into a worldwide public health concern [1,2]. As a leading cause of mortality globally, it not only triggers an array of cardiovascular and cerebrovascular disorders, but also impairs the structure and functionality of vital organs, including the heart, brain, and kidneys, thereby posing a substantial risk to human life and wellbeing [3,4,5].

*Salvia miltiorrhiza*, a traditional medicinal plant, has been extensively utilized in Chinese clinical practice for centuries [6]. Its dried roots and rhizomes, commonly known as danshen (*Salvia miltiorrhiza*, Lamiaceae) in traditional Chinese medicine, form the basis for over 900 pharmaceutical formulations approved by the China National Medical Products Administration, underscoring its therapeutic significance [7]. Danshen has been employed in hypertension management, with empirical evidence supporting its hypotensive effects [8]. Pharmacological investigations have elucidated that the antihypertensive activity of danshen is primarily mediated through the inhibition of an angiotensin-converting enzyme, a key regulatory enzyme within the renin-angiotensin system [9].

Danshensu, a water-soluble phenolic acid compound isolated from danshen, demonstrates significant blood-pressure-lowering effects. Preclinical studies in spontaneously hypertensive rats (SHR) demonstrate that intraperitoneal administration of danshensu significantly reduces systolic and diastolic blood pressure by up to 20%, an effect primarily attributed to enhanced vasodilation and suppression of vascular remodeling [8,10]. Notably, danshensu also exhibits potential protective effects against pulmonary arterial hypertension, potentially mediated via the TGF-β/Smad3 signaling pathway [11,12]. Despite promising pharmacological potential, the therapeutic potential of danshensu is significantly constrained by the following critical limitations: poor lipophilicity, chemical instability, proneness to oxidation, low oral bioavailability, and impaired blood–brain barrier permeability due to the catechol moiety and α-hydroxy acid structure. These inherent drawbacks collectively impede both its therapeutic development and clinical translation, necessitating strategic pharmacological modifications to address these multifaceted bioavailability challenges.

To address this challenge, our group designed and synthesized a series of danshensu derivatives by incorporating borneol and proline, an essential group of angiotensin-converting enzyme inhibitors, to exert antihypertensive activity [13]. This rational design strategy aimed to achieve synergistic antihypertensive effects through the complementary action of proline and to enhance drug delivery to the central nervous system by exploiting borneol’s blood–brain barrier-facilitating property. Following comprehensive evaluation, compound 221s (2,9) was identified as a lead candidate, demonstrating favorable blood pressure regulation. Experimental data indicate that 221s (2,9) reduces systolic and diastolic blood pressure in spontaneously hypertensive rats (SHR) by 50 mmHg and 35 mmHg, respectively. The potential function of 221s (2,9) to alleviate hypertension could be attributed to its dual action of suppressing the renin-angiotensin-aldosterone system and augmenting nitric oxide synthesis. Additionally, the compound demonstrates cardioprotective properties by enhancing cardiac cellular architecture and mitigating the progression of cardiac fibrosis in SHR, highlighting its capacity for organ protection [14,15].

These findings establish a foundation for the advancement of 221s (2,9) into a novel therapeutic agent. However, the translational journey from bioactive molecule to clinical application necessitates rigorous preclinical safety evaluation [16]. To the best of our knowledge, the toxicological profile of 221s (2,9) remains unexplored. It is well recognized that the pharmaceutical compounds undergo a complex and dynamic process in living organisms [17]. In this process, the formation and transformation of metabolites assumes particular significance, as metabolic modifications may preserve partial or complete pharmacological activity of the parent compound to induce novel bioactivities or to generate toxicological consequences, all of which critically influence therapeutic outcomes and safety profiles [18,19]. Given that 221s (2,9) exhibits promising pharmacological properties, it is imperative to conduct a toxicological assessment of 221s (2,9) in order to evaluate its potential as a drug candidate in the initial phases of research. Concurrently, elucidating the in vivo metabolic pathways of 221s (2,9) will provide critical insights into the observed toxicity. The findings from this study will provide a pivotal understanding for guiding rational clinical applications and informing strategic decisions in the developmental pipeline of 221s (2,9).

## 2. Results

### 2.1. Acute Toxicity of 221s (2,9)

#### 2.1.1. Determination of LD_50_

No mortality was documented in mice after receiving three distinct dosage levels of 221s (2,9) (2000, 1320, and 871.2 mg/kg) via oral gavage for seven consecutive days. Postmortem gross examination of major visceral organs revealed no macroscopic pathological abnormalities. The absence of dose-dependent toxicity precluded the calculation of LD_50_, necessitating the implementation of a maximum tolerated dose (MTD) assessment under the experimental conditions.

#### 2.1.2. Determination of MTD

The 221s (2,9) group administered 3000 mg/kg exhibited transient behavioral alterations characterized by 2 h postdosing hypoactivity (manifested as reduced locomotor engagement and quiescent states), followed by spontaneous normalization, with absence of prototypical toxicity indicators including piloerection or postural abnormalities. All experimental animals survived throughout the 7-day chronic dosing protocol, with terminal necropsy revealing equivalent organ integrity in cardiac, hepatic, and renal parenchymal tissues between the 221s (2,9)-treated and control groups. Histological examination confirmed the absence of macroscopic lesions or morphological deviations in critical visceral organs. All of these collective findings demonstrate that 221s (2,9) exhibits no significant acute toxicity, and it can be inferred that the MTD of 221s (2,9) is greater than 3000 mg/kg.

#### 2.1.3. Effects of 221s (2,9) on Body Weight, Food Intake, and Water Consumption in Mice

**Figure 1:**
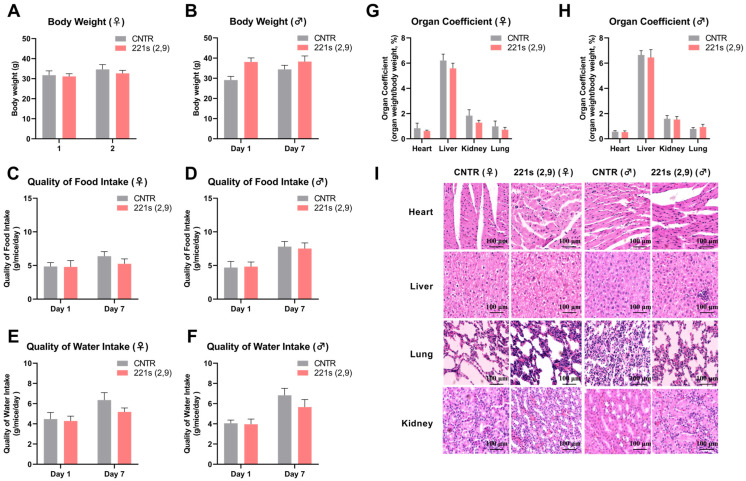
Effects of 221s (2,9) on body weight, food intake, water consumption, and pathological examination of mice in acute toxicity. (**A**) Impact on body weight of female mice. (**B**) Impact on body weight of male mice. (**C**) Impact on food intake of female mice. (**D**) Impact on food intake of male mice. (**E**) Impact on water consumption of female mice. (**F**) Impact on water consumption of male mice. (**G**) Impact on organ coefficient of female mice. (**H**) Impact on organ coefficient of male mice. (**I**) HE staining of the heart, liver, lung, and kidney of mice in CNTR and 221s (2,9) groups.

As shown in Figure 1A–F, pre-administration assessments revealed that there was no statistically significant difference in body weight, food intake, or water consumption between the control group and the 221s (2,9) treatment group. Following a 7-day intervention period with 221s (2,9) administration, comparative analysis revealed no statistically significant differences in body weight, food intake, or water consumption when evaluating the 221s (2,9) group relative to the control group, indicating that 221s (2,9) did not induce measurable alterations in these physiological metrics under the specified experimental conditions.

#### 2.1.4. Pathological Examination

Female mice in the 221s (2,9) group exhibited a modest reduction in hepatic- and splenic-mass/body-weight ratios compared to the control group (Figure 1G); moreover, these differences were not statistically significant, and these minor discrepancies were deemed clinically irrelevant to organ functionality. Comparative evaluation of other organ indices revealed minimal discrepancies between the 221s (2,9) treatment and control groups, with all measured parameters falling within the established physiological ranges for murine specimens.

The HE staining results (Figure 1I) revealed that cardiomyocytes in both male and female mice from the CNTR group exhibited well-aligned arrangements with tightly connected fibers, while female mice in the 211s (2,9) group displayed disorganized cardiac myocytes with loosened intercellular junctions. Renal HE staining further demonstrated group-specific alterations. Mice of the CNTR group and female mice of the 211s (2,9) group preserved clear glomerular structures, whereas male mice in the 211s (2,9) group exhibited loose cellular arrangements in their renal tissues. Notably, no histological differences were observed in the liver or lung tissues across all experimental groups.

### 2.2. Long-Term Toxicity of 221s (2,9)

#### 2.2.1. Effect of 221s (2,9) on the General Status of Rats

During the 12-week chronic oral administration phase and subsequent 4-week recovery observation period, all rats in the 221s (2,9)-H (80 mg/kg), 221s (2,9)-M (40 mg/kg), and 221s (2,9)-L (20 mg/kg) groups and the CNTR group exhibited normal physiological and behavioral profiles. Observations included intact and glossy fur, regular respiratory patterns (rate and depth), unimpaired limb mobility, stable mental alertness, consistent food intake, standard glandular secretion, and unremarkable urine coloration and fecal consistency. Notably, no treatment-related neurological manifestations (e.g., tremors, convulsions, ataxia) or mortality events were recorded during the entire experimental protocol.

#### 2.2.2. Effect of 221s (2,9) on Body Weight, Food Intake, and Water Consumption in Rats

**Figure 2:**
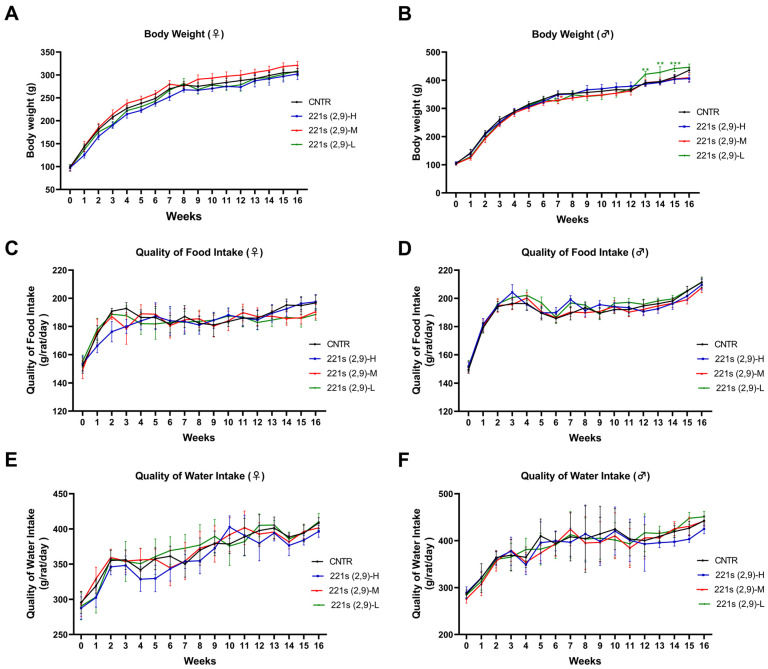
Effects of 221s (2,9) on body weight, food intake, and water consumption of rats in long-term toxicity mice. (**A**) Impact on body weight of female rats. (**B**) Impact on body weight of male rats. (**C**) Impact on food intake of female rats. (**D**) Impact on food intake of male rats. (**E**) Impact on water consumption of female rats. (**F**) Impact on water consumption of male rats. * *p* < 0.05, ** *p* < 0.01, *** *p* < 0.001 compared to CNTR.

Throughout the experimental period, all rats exhibited sustained body weight gain. Comparative analyses demonstrated no statistically significant differences in body weight between female rats in the 221s (2,9)-H (80 mg/kg), 221s (2,9)-M (40 mg/kg), and 221s (2,9)-L (20 mg/kg) groups or the CNTR group (Figure 2A). In male rats, the 221s (2,9)-M and 221s (2,9)-L groups displayed a statistically significant attenuation of weight gain during week 7 (Figure 2B). Notably, the 221s (2,9)-L male group manifested a paradoxical acceleration in weight gain during weeks 13–15 of the recovery phase, achieving marked statistical significance compared to control group.

As shown in Figure 2C–F, all experimental groups demonstrated progressive increases in water consumption and food intake within physiologically normal ranges. Furthermore, comparative analyses revealed no statistically significant variations in water consumption and food intake between the 221s (2,9)-H, 221s (2,9)-M, and 221s (2,9)-L groups and their time- and sex-matched vehicle control group.

#### 2.2.3. Hematology Analysis

As shown in Table 1, compared with the control group, female rats administered with 221s (2,9) (40 mg/kg) for 12 weeks exhibited significantly reduced white blood cell counts (*p* < 0.01), neutrophil counts (*p* < 0.05), and lymphocyte counts (*p* < 0.01), with all parameters returning to normal levels in following 4 weeks. Compound 221s (2,9) demonstrated slight immunomodulatory effects in female rats, as evidenced by elevated monocyte percentages in the 221s (2,9)-M (*p* < 0.01) and 221s (2,9)-L (*p* < 0.01) groups, along with increased mean corpuscular hemoglobin (MCH) levels in the 221s (2,9)-M (*p* < 0.01) and 221s (2,9)-L (*p* < 0.01) groups. However, these fluctuations remained within physiological reference ranges and did not exhibit a dose–effect relationship, leading to the conclusion that these observations lack toxicological significance.

No statistically significant differences in hematological parameters were observed between any dose groups of male rats treated with 221s (2,9) for 12 weeks and the control group (Table 2).

#### 2.2.4. Biochemistry Analysis

**Figure 3:**
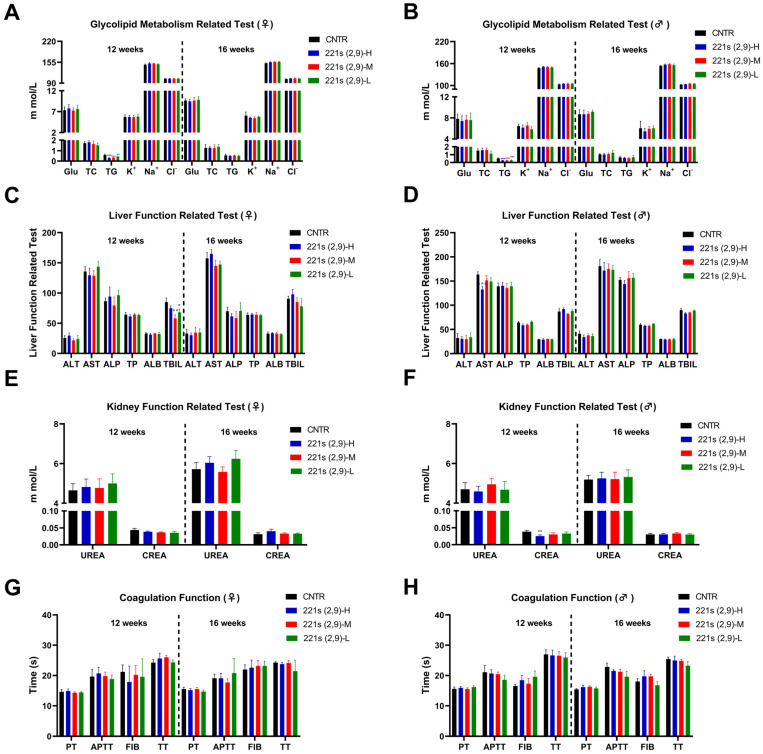
Effects of 221s (2,9) on biochemical parameters of rats in long-term toxicity mice. (**A**) Impact on liver function of female rats. (**B**) Impact on liver function of male rats. (**C**) Impact on kidney function of female rats. (**D**) Impact on kidney function of male rats. (**E**) Impact on coagulation function of female rats. (**F**) Impact on coagulation function of male rats. (**G**) Impact on glycolipid metabolism of female rats. (**H**) Impact on glycolipid metabolism of male rats. * *p* < 0.05, ** *p* < 0.01, *** *p* < 0.001 compared to CNTR.

As shown in Figure 3A,B, compared with the control group, the 221s (2,9)-treated groups of both female and male rats exhibited significant reductions in TG levels (*p* < 0.01), indicating that 221s (2,9) possesses hypolipidemic effects.

Notably, female rats in 221s (2,9)-M manifested a 31.8% reduction in serum total bilirubin (TBIL) levels versus the controls (*p* < 0.01) (Figure 3C), while other hepatic parameters, including ALT, AST, and ALP, remained unaffected, demonstrating no pathological relevance. Comprehensive analysis confirmed that all values of additional biochemical markers relating to kidney function (Figure 3E) and coagulation function (Figure 3G) in female 221s (2,9)-treated groups shows statistically indistinguishable results from those of the baseline controls.

In male rats, the 221s (2,9)-H group manifested a transient 34.2% reduction in serum creatinine (CREA) that demonstrated neither dose dependency nor post-recovery persistence, confirming its pharmacological irrelevance (Figure 3F). Additionally, the 221s (2,9)-L male group displayed reduced K+ ion concentrations (*p* < 0.05), while elevated Na+ ion concentrations were detected in the 221s (2,9)-H and 221s (2,9)-M groups. All values fluctuated within physiological reference ranges without clinically significant deviations, indicating statistical but no toxicological relevance. Comprehensive analysis confirmed that all values of additional biochemical markers relating to liver function (Figure 3D) and coagulation function (Figure 3H) in male 221s (2,9)-treated groups showed statistically indistinguishable results from those of the baseline controls.

#### 2.2.5. Pathological Examination

**Figure 4:**
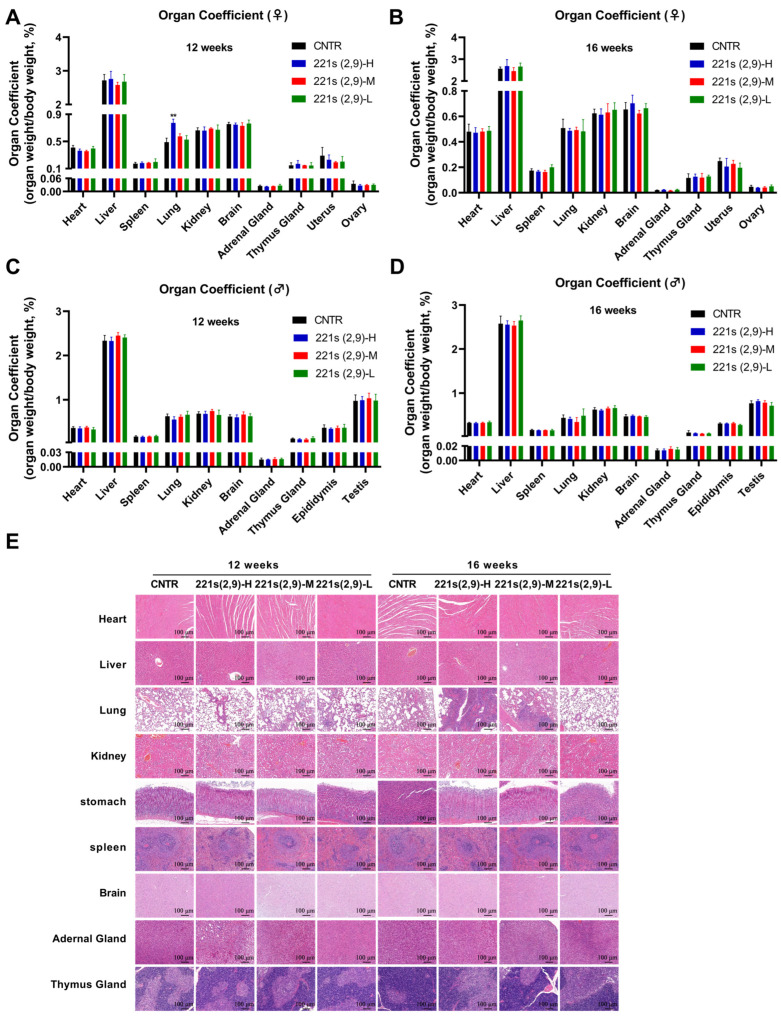
Effects of 221s (2,9) on pathological examination of rats in long-term toxicity mice. Pathological examination: (**A**) Impact on organ coefficient of main organs of female rats in 12 weeks of 221s (2,9) treatment. (**B**) Impact on organ coefficient of main organs of female rats in a 4-week recovery period after 12 weeks of 221s (2,9) treatment. (**C**) Impact on organ coefficient of main organs of male rats in 12 weeks of 221s (2,9) treatment. (**D**) Impact on organ coefficient of main organs of male rats in a 4-week recovery period after 12 weeks of 221s (2,9) treatment. (**E**) HE staining of main organs. ** *p* < 0.01.

As illustrated in Figure 4A, in comparison to the CNTR group, female rats of the 221s (2,9)-H group exhibited a marked elevation in lung coefficient values (*p* < 0.01). Moderate consistent elevations were observed in another two treated groups, demonstrating a discernible dose–response relationship. These findings suggest potential chronic pulmonary toxicity associated with prolonged 221s (2,9) administration in female subjects. Following a 4-week drug withdrawal recovery period (Figure 4B), no significant difference in organ coefficients was observed between female rats of the treatment groups and the CNTR group (*p* > 0.05), indicating reversible pulmonary alterations. The male rats exhibited no statistically significant variations in organ coefficients between the treatment groups and the CNTR group across all tested dosages (*p* > 0.05). The HE staining results revealed no drug-associated histopathological abnormalities in cardiac, hepatic, splenic, pulmonary, or renal tissues of rats in the 221s (2,9)-treated groups.

### 2.3. Metabolites and Metabolic Pathway of 221s (2,9)

#### 2.3.1. Mass Fragmentation of 221s (2,9)

**Figure 5:**
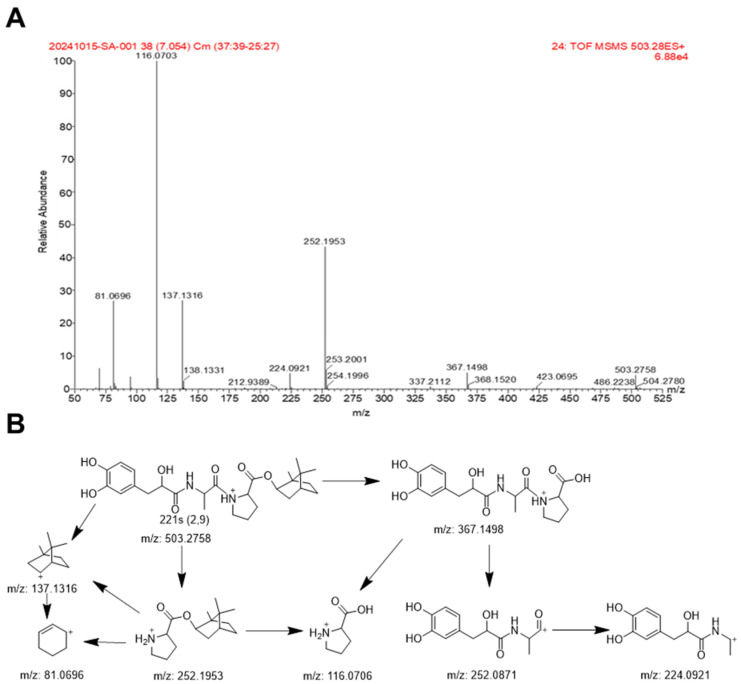
MS/MS spectrum (**A**) and proposed mass fragmentation pathways (**B**) of 221s (2,9) in positive ion mode.

The mass fragmentation of 221s (2,9) was analyzed to identify the metabolites effectively. In positive ion mode, the retention time of 221s (2,9) is 7.09 min, with a protonated molecular ion peak at *m*/*z* 503.2758 [M+H]^+^ with a molecular formula of C_27_H_38_N_2_O_7_. The mass error between the experimental and theoretical mass was −0.0042 ppm. The product ion mass spectrum of 221s (2,9) is illustrated in Figure 5A, including *m*/*z* 367.1496, 252.1953, 252.0871, 224.0921, 137.1316, 116.0703, and 81.0696. The carboxamide bond (CO-NH) cleavage of molecular ion resulted in a fragment of *m*/*z* of 252.1953 (M-C_12_H_13_NO_5_) and 252.0871 (M-C_15_H_25_NO_2_) due to the loss of C_12_H_13_NO_5_ and C_27_H_38_N_2_O_7_, respectively. The ion of *m*/*z* of 252.1953 was fragmented into *m*/*z* of 137.1316 (M-C_12_H_13_NO_5_-C_5_H_9_NO_2_) and 116.0706 (M-C_12_H_13_NO_5_-C_10_H_16_) due to putative loss of C_5_H_9_NO_2_ and C_10_H_16_, respectively. The ion of *m*/*z* of 137.1316 was further fragmentated into *m*/*z* of 81.0696 (M-C_12_H_13_NO_5_-C_5_H_9_NO_2_-C_4_H_8_) by the loss of C_4_H_8_. Another two characteristic fragment ions were observed after the ester bond (CO-O) cleavage, resulting in a fragment of *m*/*z* of 137.1316(M-C_17_H_20_N_2_O_6_-H_2_O) and 367.1498(M-C_10_H_16_), which generated the daughter ion of *m*/*z* of 252.0871(M-C_10_H_16_-C_5_H_9_NO_2_) and 116.0706(M-C_10_H_16_-C_12_H_13_NO_5_) due to the loss of C_5_H_9_NO_2_ and C_12_H_13_NO_5_. The ion of *m*/*z* of 252.0871, generated the daughter ion of *m*/*z* of 224.0921(M-C_10_H_16_-C_5_H_9_NO_2_-CO) due to loss of CO. 221s (2,9), readily forms fragment ion peaks by the cleavage of the ester bond and carboxamide bond and the loss of the CO and alkane group, which may be related to the presence of ester groups, a carboxamide group, aliphatic chains, and hydroxyl groups in its molecular structure. The possible fragmentation mechanism of 221s (2,9) is presented in Figure 5B. Notably, the signal for *m*/*z* of 252.0871 was not observed under our analytical conditions; however, it is included in the proposed pathway based on common fragmentation patterns and its consistent presence in metabolite studies, serving as a plausible precursor to the fragment at *m*/*z* of 224.0921.

Since most metabolites retain the core structure of 221s (2,9), the mass spectrometry fragmentation patterns obtained above can serve as a reference for the structural identification of subsequent metabolites.

#### 2.3.2. Identification of the Metabolites of 221s (2,9)

**Figure 6:**
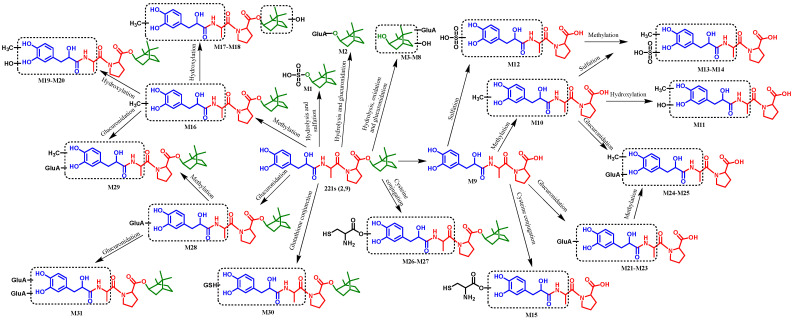
The possible metabolic pathways of 221s (2,9) in rats.

A total of 31 metabolites of 221s (2,9) generated through Phase I and II reaction were detected, including 16 metabolites in the plasma, 9 metabolites in the urine, 19 metabolites in the bile, and 16 metabolites in the feces. The relevant information about the metabolites is shown in Table 3, and the main metabolic pathways are illustrated in Figure 6.

#### 2.3.3. Structure Elucidation of the Metabolites of 221s (2,9)

The molecular ion of metabolite M0 was at *m*/*z* 503.2758, with a retention time (RT) at 7.08 min. The MS/MS spectrum showed the product ions at *m*/*z* 367.1498, 252.1953, 116.0703, and 137.1316, which were consistent with the mass fragmentation of 221s (2,9), indicating that M0 was the parent compound 221s (2,9).

Metabolite M1, detected at 3.73 min, produced the molecular ion [M+H]^+^ at *m*/*z* 235.0992, with an assigned elemental composition of C_10_H_19_O_4_S^+^. The characteristic fragment ion peaks of metabolite M1 were at *m*/*z* 155.1459, 137.1304, and 81.0689. The characteristic fragment ion at *m*/*z* 155.1459(M-SO_3_) was produced to lose 80 Da (SO_3_) from M1, as suggested by the existence of sulfonate. The product ion at *m*/*z* 137.1304 (M-H_2_O -SO_3_), which was the characteristic fragment ion of 221s (2,9), was produced to lose 18 Da (H_2_O) from the ion at *m*/*z* 155.1459, indicating that hydrolysis occurred. Therefore, it could be deduced that the proposed metabolite M1 was the hydrolysis and sulfation product of 221s (2,9).

Metabolite M2, detected at 5.60 min, produced the molecular ion [M+H]^+^ at *m*/*z* 331.1748, with an assigned elemental composition of C_16_H_27_O_7_^+^. The characteristic fragment ion peaks of metabolite M2 were at *m*/*z* 155.1463, 137.1306, and 81.0713, which were consistent with the fragmentation pathways of metabolite M1, suggesting that hydrolysis occurred. The characteristic fragment ion at *m*/*z* 155.1463 (M-C_6_H_8_O_6_) was produced to lose 176 Da (C_6_H_8_O_6_) from metabolite M2, as suggested by the existence of glucuronidation. Therefore, metabolite M2 was deduced as the hydrolysis and glucuronidation product of 221s (2,9).

Metabolites M3, M4, M5, M6, M7, and M8 were detected at 3.55, 3.74, 4.01, 4.35, 4.61, and 4.91 min, respectively. They possessed protonated molecular ions at 347.1713, 347.1712, 347.1708, 347.1707, 347.1694, and 347.1728, with an assigned elemental composition of C_16_H_27_O_8_^+^, which were 16 Da higher than M2, implying that oxidation reaction occurred. The characteristic fragment ion at *m*/*z* 171.1455 (M-C_6_H_8_O_6_) was produced to lose 176 Da (C_6_H_8_O_6_) from metabolite M3, suggesting the existence of glucuronidation. The other product ions at *m*/*z* 155.1461 (M-C_6_H_8_O_6_-O) and 137.1305 (M-C_6_H_8_O_6_-O-H_2_O) were identical to those of metabolite M2. Therefore, metabolite M3 was deduced as the hydrolysis, oxidation, and glucuronidation product of 221s (2,9). Metabolites M4, M5, M6, M7, and M8, which possessed the same fragment ions as M3, were regarded as the isomers of M3.

Metabolite M9 was formed due to the sensitivity of the ester bond in the molecule to hydrolysis. It can be detected in samples of plasma, urine, and feces with an RT of 3.46 min. As illustrated in Table 3, metabolite M9 produced the molecular ion [M+H]^+^ at *m*/*z* 367.1493, which was the fragment ion of M0 after the eater bond cleavage. The fragment ion peaks of M9 were at *m*/*z* 252.0880 (M-C_5_H_9_NO_2_), 224.0908 (M-C_5_H_9_NO_2_-CO), and 116.0696 (M-C_12_H_13_NO_5_), which were consistent with the fragmentation pathways of M0, indicating that M9 was the ester bond hydrolysis product of 221s (2,9) after losing the borneol structure.

Metabolite M10 can be detected at 3.88 min, exhibiting a molecular ion peak [M+H]^+^ at 381.1627, with an assigned elemental composition of C_18_H_25_N_2_O_7_^+^, which was 14 Da higher than those of M9. Metabolite M10 generated product ions at *m*/*z* 266.1018, 238.1068, 116.0696, and 70.0640. The fragment ions of *m*/*z* 266.1018 (M-C_5_H_9_NO_2_) and 238.1068 (M-C_5_H_9_NO_2_-CO) were 14 Da more than those of metabolite M9, implying that it is a methylated product. The fragment ions of *m*/*z* 116.0696 (M-CH_2_-C_12_H_13_NO_5_) and 70.0640 (M-CH_2_-C_12_H_13_NO_5_-CH_2_O_2_) were identical to those of M9, suggesting that the methylation site is located on the benzene ring side. Therefore, metabolite M10 was deduced as the hydrolysis and methylation product of 221s (2,9).

Metabolite M11 was detected at 3.11 min, exhibiting a molecular ion peak [M+H]^+^ at 397.1597, with an assigned elemental composition of C_18_H_25_N_2_O_8_^+^, which was 16 Da higher than those of M10 and 30 Da higher than those of M9, indicating that methylation and oxidation reaction occur. Metabolite M11 generated product ions at *m*/*z* 282.0818, 254.0692, 116.0537, and 70.0637. The fragment ions of *m*/*z* 282.0818 (M-C_5_H_9_NO_2_) and 254.0692 (M-C_5_H_9_NO_2_-CO) were 30 Da more than those of metabolite M9 and 16 Da more than those of metabolite M10, and the fragment ions of *m*/*z* 116.0696 (M-CH_2_-O-C_12_H_13_NO_5_) and 70.0640 (M-CH_2_-O-C_12_H_13_NO_5_-CH_2_O_2_) were identical to those of M9 and M10, suggesting that the methylation and oxidation sites were located on the group of danshensu or the linked alanine. Therefore, metabolite M11 was deduced as the hydrolysis, methylation, and oxidation product of 221s (2,9).

Metabolite M12 was detected at 3.48 min, exhibiting a molecular ion peak [M+H]^+^ at 447.1069, with an assigned elemental composition of C_18_H_25_N_2_O_10_S^+^, which was 80 Da (SO_3_) higher than those of M9 (the hydrolysis product of 221s (2,9)), indicating that sulfation reaction occurs. Metabolite M12 generated product ions at *m*/*z* of 367.1480, 332.0825, 252.0854, 116.0627, and 70.0653. The fragment ions of *m*/*z* 367.1480 (M-SO_3_) and 332.0825 (M-SO_3_-C_5_H_9_NO_2_) were 80 Da more than those of metabolite M9, and the fragment ions of *m*/*z* 116.0696 (M-SO_3_-C_12_H_13_NO_5_) and 70.0640 (M-SO_3_-C_12_H_13_NO_5_-CH_2_O_2_) were identical to those of M9, further suggesting that the sulfonation sites were located on the group of danshensu or the linked alanine. Therefore, metabolite M12 was deduced as the hydrolysis and sulfation product of 221s (2,9).

Metabolites M13 and M14 were detected at 3.55 and 3.90 min, respectively. They possessed protonated molecular ions at 461.1256 and 461.1245, with an assigned elemental composition of C_18_H_25_N_2_O_10_S^+^, which was 80 Da (SO_3_) higher than those of M10 and 14 Da (CH_2_) higher than those of M12, implying that methylation and sulfonate reactions occurred. Metabolite M13 generated product ions at *m*/*z* 381.1643, 318.0637, 238.1075, 116.0693, and 70.0635. The product ions at *m*/*z* 116.0693 (M-SO_3_-CH_2_-C_12_H_13_NO_5_) and 70.0635 (M-SO_3_-CH_2_-C_12_H_13_NO_5_-CH_2_O_2_) were identical to those of 221s (2,9), and the product ions at *m*/*z* 238.1075 (M-SO_3_-C_5_H_9_NO_2_-CO) were 14 Da (CH_2_) higher than those of 221s (2,9) (*m*/*z* 224.0921), suggesting that the methylation site occurred on the danshensu group or the linked amide group. The characteristic fragment ions at *m*/*z* 381.1643 (M-SO_3_) and 238.1075 (M-SO_3_-C_5_H_9_NO_2_-CO) were produced to lose 80 Da (SO_3_) from *m*/*z* 461.1256 and 318.0637 (M-C_5_H_9_NO_2_-CO), indicating that the sulfation site occurred on the danshensu group or the linked amide group. Therefore, metabolite M13 was deduced as the hydrolysis, methylation, and sulfation product of 221s (2,9). M14, which possessed the same fragment ions as M13, was regarded as the isomer of M13.

Metabolite M15 was detected at 3.09 min, exhibiting a molecular ion peak [M+H]^+^ at 486.1546, with an assigned elemental composition of C_20_H_28_N_3_O_9_S^+^, which was 119 Da (C_3_H_5_NO_2_S) higher than those of M9 (the hydrolysis product of 221s (2,9)), indicating that a cysteine conjunction reaction occurred. Metabolite M15 generated product ions at *m*/*z* 371.0909, 343.0982, 308.0583, 280.0638, 210.0206, 116.0703, and 70.0637. The fragment ions of *m*/*z* 371.0909 (M-C_5_H_9_NO_2_) and 343.0982 (M-C_5_H_9_NO_2_-CO) were 119 Da more than those of metabolite M9, and the fragment ions of *m*/*z* 116.0703 (M-C_3_H_5_NO_2_S-C_12_H_13_NO_5_) and 70.0637 (M-C_3_H_5_NO_2_S-C_12_H_13_NO_5_-CH_2_O_2_) were identical to those of M9, further suggesting that the sites of cysteine conjunction were located on the side of the danshensu group or the linked amide group. Therefore, metabolite M15 was deduced as the hydrolysis and cysteine conjunction product of 221s (2,9).

Metabolite M16 was detected at 7.44 min, exhibiting a molecular ion peak [M+H]^+^ at 517.2914, with an assigned elemental composition of C_28_H_41_N_2_O_7_^+^, which was 14 Da higher than those of 221s (2,9), indicating that a methylation reaction occurred. Metabolite M16 generated product ions at *m*/*z* 381.1663, 266.1040, 252.1957, 238.1068, 137.1319, 116.0700, and 81.0697. The fragment ions of *m*/*z* 381.1663 (M-C_10_H_16_), 266.1040 (M-C_10_H_16_-C_5_H_9_NO_2_), and 238.1068(M-C_10_H_16_-C_5_H_9_NO_2_-CO) were 14 Da more than those of 221s (2,9), and the fragment ions of *m*/*z* 137.1319 (M-CH_2_-C_17_H_20_N_2_O_6_-H_2_O), 116.0700 (M-CH_2_-C_10_H_16_-C_12_H_13_NO_5_), and 81.0697 (M-CH_2_-C_12_H_13_NO_5_-C_5_H_9_NO_2_-C_4_H_8_) were identical to those of 221s (2,9), further suggesting that the sites of methylation reaction were located on the side of the danshensu group or the linked amide group. Therefore, metabolite M16 was deduced as the methylation product of 221s (2,9).

Metabolites M17, M18, M19, and M20 were detected at 5.05, 5.65, 7.02, and 7.10 min, respectively. They possessed protonated molecular ions at 533.2901, 533.2884, 533.2888, and 533.2895, with an assigned elemental composition of C_28_H_41_N_2_O_8_^+^, which was 30 Da higher than those of 221s (2,9) and 16 Da higher than those of M16, implying the methylation and hydroxylation reactions occurred. Metabolite M17 generated product ions at *m*/*z* 492.1459, 422.8778, 381.1678, 268.1917, and 116.0706. The product ion at *m*/*z* of 116.0693 (M-CH_2_-O-C_10_H_16_-C_12_H_13_NO_5_) was identical to those of 221s (2,9), and the product ion at *m*/*z* of 268.1917 (M-CH_2_-C_12_H_13_NO_5_) was 16 Da higher than those of 221s (2,9) (*m*/*z* 252.1953), suggesting that the hydroxylation site occurred on the borneol group. The characteristic fragment ion at *m*/*z* 381.1678 (M-O-C_10_H_16_) was 14 Da higher than that of 221s (2,9) (*m*/*z* 367.1498), indicating that the methylation site occurred on the danshensu group or the linked amide group. Metabolite M18 was regarded as the isomer of M17 for revealing the same fragment ions, indicating that they possessed similar methylation and hydroxylation sites. Metabolite M19 generated product ions at *m*/*z* 397.1460, 252.1971, and 116.0703. The product ions at *m*/*z* of 116.0693 (M-CH_2_-O-C_10_H_16_-C_12_H_13_NO_5_) and 252.1971 (M-CH_2_-O-C_12_H_13_NO_5_) were identical to those of 221s (2,9), and the product ion at *m*/*z* of 397.1460 (M-C_10_H_16_) was 30 Da higher than those of 221s (2,9) (*m*/*z* 367.1498) and 16 Da higher than that of M16 (*m*/*z* 381.1663), demonstrating that methylation and hydroxylation sites occurred on the danshensu group or the linked amide group. M20 was regarded as the isomer of M19 for revealing the same fragment ions, indicating that it possessed similar methylation and hydroxylation sites. Therefore, metabolites M17, M18, M19, and M20 were deduced as the methylation and hydroxylation products of 221s (2,9).

Metabolites M21, M22, and M23 were detected at 2.95, 3.33, and 4.15 min, respectively. They possessed protonated molecular ions at 543.1838, 543.1807, and 543.1815, with an assigned elemental composition of C_23_H_31_N_2_O_13_^+^, which was 176 Da (C_6_H_8_O_6_) higher than those of metabolite M9, indicating the glucuronidation conjunction occurred. Metabolite M21 generated product ions at *m*/*z* 367.1484, 252.0841, 224.0886, 116.0688, and 70.0620. The product ions at *m*/*z* of 252.0841 (M-C_6_H_8_O_6_-C_10_H_16_-C_5_H_9_NO_2_), 224.0886 (M-C_6_H_8_O_6_-C_10_H_16_-C_5_H_9_NO_2_-CO), 116.0688 (M-C_6_H_8_O_6_-C_10_H_16_-C_12_H_13_NO_5_), and 70.0620 (M-C_6_H_8_O_6_-C_10_H_16_-C_12_H_13_NO_5_-CH_2_O_2_) were identical to those of 221s (2,9), and the characteristic fragment ion at *m*/*z* 367.1484 (M-C_6_H_8_O_6_-C_10_H_16_) was produced to lose 176 Da (-C_6_H_8_O_6_) from *m*/*z* of 543.1838, as suggested by the existence of glucuronidation. Metabolites M22 and M23 were regarded as the isomers of M20 for revealing the same fragment ions. Therefore, metabolites M21, M22, and M23 were deduced as the hydrolysis and glucuronidation conjunction products of 221s (2,9).

Metabolites M24 and M25 were detected at 3.38 and 3.60 min, respectively. They possessed protonated molecular ions at 557.1971 and 557.1973, with an assigned elemental composition of C_24_H_33_N_2_O_13_^+^, which was 14 Da higher than those of metabolite M21 (the hydrolysis and glucuronidation conjunction product of 221s (2,9)), indicating that methylation reaction might exist. Metabolite M24 generated product ions at *m*/*z* 442.1329, 381.1643, 266.1020, 238.1037, and 116.0690. The product ion at *m*/*z* of 116.0693 (M-C_6_H_8_O_6_-CH_2_-C_10_H_16_-C_12_H_13_NO_5_) was identical to those of 221s (2,9), and the product ions at *m*/*z* of 266.1020 (M-C_6_H_8_O_6_-C_10_H_16_-C_5_H_9_NO_2_) and 238.1037 (M-C_6_H_8_O_6_-C_10_H_16_-C_5_H_9_NO_2_-CO) were 14 Da higher than those of 221s (2,9) (*m*/*z* of 252.0871 and 224.0921), demonstrating that methylation sites occurred on the danshensu group or the linked amide group. The characteristic fragment ion at *m*/*z* 266.1020 (M-C_6_H_8_O_6_-C_10_H_16_-C_5_H_9_NO_2_) was produced to lose 176 Da (-C_6_H_8_O_6_) from *m*/*z* of 442.1329 (M-C_10_H_16_-C_5_H_9_NO_2_), as suggested by the existence of glucuronidation with the conjunction site located on the danshensu group or the linked amide group. Metabolite M25 was regarded as the isomer of M24 for showing the same fragment ions. Therefore, metabolites M24 and M25 were deduced as the hydrolysis, methylation, and glucuronidation conjunction products of 221s (2,9).

Metabolites M26 and M27 were detected at 6.10 and 6.22 min, respectively. They possessed protonated molecular ions at 622.2793 and 622.2813, with an assigned elemental composition of C_30_H_44_N_3_O_9_S+, which was 119 Da (C_3_H_5_NO_2_S) higher than those of metabolite 221s (2,9), indicating that a cysteine conjunction reaction might exist. Metabolite M26 generated product ions at *m*/*z* of 486.1555, 371.0884, 325.0864, 252.0950, 116.0697, and 81.0687. The product ions at *m*/*z* 252.1950 (M-C_3_H_5_NO_2_S-C_10_H_16_-C_5_H_9_NO_2_), 116.0693 (M-C_3_H_5_NO_2_S-C_10_H_16_-C_12_H_13_NO_5_), and 81.0687 (M-C_3_H_5_NO_2_S-C_12_H_13_NO_5_-C_5_H_9_NO_2_-C_4_H_8_) were identical to those of 221s (2,9), and the product ion at *m*/*z* of 371.0884 (M-C_10_H_16_-C_5_H_9_NO_2_) was 119 Da (C_3_H_5_NO_2_S) higher than that of metabolite 221s (2,9) (*m*/*z* of 252.0871), demonstrating that the site of cysteine conjunction occurred on the danshensu group or the linked amide group. M27, which possessed the same fragment ions as M26, was regarded as the isomer of M26. Therefore, metabolites M26 and M27 were deduced as the cysteine conjunction product of 221s (2,9).

Metabolite M28 was detected at 6.56 min, exhibiting a molecular ion peak [M+H]^+^ at 679.3076, with an assigned elemental composition of C_33_H_47_N_2_O_13_^+^, which was 176 Da (C_6_H_8_O_6_) higher than those of 221s (2,9), indicating the glucuronidation conjunction occurred. Metabolite M28 generated product ions at *m*/*z* 503.2747, 428.1180, 367.1491, 252.0944, 224.0898, 116.0694 and 81.0683. The fragment ions of *m*/*z* 503.2747 (M-C_6_H_8_O_6_), 367.1491 (M-C_6_H_8_O_6_-C_10_H_16_), 252.1944 (M-C_6_H_8_O_6_-C_12_H_13_NO_5_), 224.0898 (M-C_6_H_8_O_6_-C_10_H_16_-C_5_H_9_NO_2_-CO), 116.0694 (M-C_6_H_8_O_6_-C_10_H_16_-C_12_H_13_NO_5_) and 81.0683 (M-C_6_H_8_O_6_-C_12_H_13_NO_5_-C_5_H_9_NO_2_-C_4_H_8_) were identical to those of 221s (2,9) and the fragment ion at *m*/*z* 252.1944 (M-C_6_H_8_O_6_-C_12_H_13_NO_5_) was produced to lose 176 Da (C_6_H_8_O_6_) from characteristic fragment ion at *m*/*z* 543.1838 (M-C_12_H_13_NO_5_), as suggested by glucuronidation conjunction were located on the side of the danshensu group or the linked amide group. Therefore, metabolite M28 was deduced as the glucuronidation conjunction product of 221s (2,9).

Metabolite M29 was detected at 6.63 min, exhibiting a molecular ion peak [M+H]^+^ at 693.3259, with an assigned elemental composition of C_34_H_49_N_2_O_13_^+^, which was 14 Da higher than that of metabolite M28, indicating the glucuronidation conjunction and methylation reaction might occur. Metabolite M29 generated product ions at *m*/*z* 517.2813, 442.1249, 381.1685, 266.1019, 252.1945, 116.0690 and 81.0678. The fragment ion at *m*/*z* 517.2813 (M-C_6_H_8_O_6_) was produced to lose 176 Da (C_6_H_8_O_6_) from the metabolite M29, further confirming the occurrence of the glucuronidation conjunction reaction. The fragment ions of *m*/*z* 442.1249 (M-C_6_H_8_O_6_-C_10_H_16_) and 266.1018 (M-C_6_H_8_O_6_-C_12_H_13_NO_5_) were 14 Da higher than that of metabolite M28, suggesting that the methylation and glucuronidation conjunction were located on the side of the danshensu group or the linked amide group. Therefore, metabolite M29 was deduced as the methylation and glucuronidation conjunction product of 221s (2,9).

Metabolite M30 was detected at 6.02 min, exhibiting a molecular ion peak [M+H]^+^ at 808.3371, with an assigned elemental composition of C_37_H_54_N_5_O_13_S+, which was 305 Da (C_10_H_15_N_3_O_6_S) higher than those of 221s (2,9), indicating the glutathione conjunction might occur. Metabolite M30 generated product ions at *m*/*z* 672.3594, 557.1544, 252.1939, and 81.0700. The product ions at *m*/*z* 252.1939 (M-C_10_H_15_N_3_O_6_S-C_12_H_13_NO_5_) and 81.0700 (M-C_10_H_15_N_3_O_6_S-C_12_H_13_NO_5_-C_5_H_9_NO_2_-C_4_H_8_) were identical to those of 221s (2,9), and fragment ions of *m*/*z* 672.3594 (M-C_10_H_16_) and 557.1544 (M-C_10_H_16_-C_5_H_9_NO_2_) were 305 Da higher than those of 221s (2,9), suggesting that the glutathione conjunctions were located on the side of the danshensu group or the linked amide group. Therefore, metabolite M30 was deduced as the glutathione conjunction product of 221s (2,9).

Metabolite M31 was detected at 6.19 min, exhibiting a molecular ion peak [M+H]^+^ at 855.3372, with an assigned elemental composition of C_39_H_55_N_2_O_19_^+^, which was 352 Da higher than those of 221s (2,9), indicating that bi-glucuronidation conjunction might occur. Metabolite M31 generated product ions at *m*/*z* 679.3068, 543.1871, 503.2729, 428.1123, 367.1534, 252.1949, and 116.0699. The fragment ion at *m*/*z* 503.2729 (M-C_6_H_8_O_6_-C_6_H_8_O_6_) was produced to lose 176 Da (C_6_H_8_O_6_) from the characteristic fragment ion at *m*/*z* 679.3068 (M-C_6_H_8_O_6_), which was the production of metabolite M31 by the elimination of 176 Da. The fragment ions of *m*/*z* 543.1871 (M-C_6_H_8_O_6_-C_10_H_16_) and 428.1123 (M-C_6_H_8_O_6_-C_10_H_16_-C_5_H_9_NO_2_) were 176 Da higher than those of 221s (2,9) (*m*/*z* of 367.1498 and 252.0871), and the product ions at *m*/*z* 367.1534 (M-C_6_H_8_O_6_-C_6_H_8_O_6-_C_10_H_16_), 252.1949 (M-C_6_H_8_O_6_-C_6_H_8_O_6_-C_12_H_13_NO_5_), and 116.0699 (M-C_6_H_8_O_6_-C_6_H_8_O_6-_C_10_H_16_-C_12_H_13_NO_5_) were identical to those of 221s (2,9), suggesting that the glucuronidation conjunction might be located on the side of the danshensu group or the linked amide group. Therefore, metabolite M31 was deduced as the bi-glucuronidation conjunction product of 221s (2,9).

#### 2.3.4. Metabolic Pathway of 221s (2,9)

The proposed metabolic pathways of 221s (2,9) are illustrated in Figure 4, demonstrating that 221s (2,9) undergoes a series of metabolic reactions, including Phase I reactions such as hydrolysis, hydroxylation, oxidation, and methylation, and Phase II conjugations, such as glucuronidation, cysteine conjunction, and glutathione conjunction. Additionally, 221s (2,9) exhibits a propensity to form conjugated metabolites, including methylation, glucuronidation, and hydroxylation derivatives, a pattern analogous to the metabolic profile of danshensu in vivo [20].

## 3. Discussion

As a foundational herb in traditional Chinese medicine, *Salvia miltiorrhiza* has been employed for millennia in managing cardiovascular conditions [21]. Danshensu, a principal water-soluble bioactive constituent, has attracted significant research focus due to its angiotensin-converting enzyme (ACE) inhibitory activity and endothelial protective functions [22]. Nevertheless, the clinical advancement of danshensu remains severely constrained by an unfavorable pharmacokinetic profile, exhibiting notably low oral bioavailability, rapid metabolic clearance, and restricted blood–brain barrier penetration. These inherent limitations necessitated rational structural modification. Compound 221s (2,9), a novel danshensu-derived antihypertensive agent, exhibits superior antihypertensive efficacy and multifaceted cardiovascular protective effects in SHR rats.

Toxicity evaluation constitutes a critical component of pharmaceutical development [23]. In this study, we systematically conducted acute and long-term toxicity studies on a novel danshensu-derived antihypertensive agent, 221s (2,9), combined with UPLC-MS/MS-based identification of 31 metabolites, providing critical evidence to support clinical translation.

In the acute toxicity study, single oral administration of 221s (2,9) to mice at doses of 871.2, 1320, and 2000 mg/kg resulted in no mortality or adverse clinical signs. The LD50 could not be determined due to the absence of dose-dependent lethality, prompting the evaluation of MTD. At the maximal administrable concentration of 3000 mg/kg via oral gavage, neither mortality nor behavioral abnormalities were observed in the treatment groups. During the 7-day observation period, no statistically significant differences were detected in body weight fluctuations, food/water consumption, or vital organ indices between the groups. These findings collectively establish the MTD of 221s (2,9) as 3000 mg/kg, with no evident acute toxicity manifestations under experimental conditions.

According to the long-term toxicity assessment in SD rats, 221s (2,9) is safe when given for 12 consecutive weeks at three dosage levels under standardized experimental conditions. Hematological parameters derived from animal studies provide a basis for predicting adverse effects in clinical settings. The long-term toxicity assessment revealed sex-specific and generally reversible alterations in hematological and biochemical parameters following 12-week 221s (2,9) administration. In female rats, transient reductions in leukocyte subpopulations (neutrophils, lymphocytes) at 40 mg/kg were fully normalized post-recovery, suggesting adaptive rather than cytotoxic responses. Compound 221s (2,9) possesses hypolipidemic activity, evidenced by sustained TG reduction in both sexes. Notably, the 31.8% TBIL decrease in mid-dose females lacked correlative hepatocyte injury markers (ALT/AST/ALP stability) or histopathological evidence, implying indirect bilirubin metabolism modulation rather than hepatotoxicity. Similarly, transient creatinine fluctuations in high-dose males and electrolyte variations (K^+^/Na^+^) across groups were pharmacologically insignificant for a non-persistent nature. Notably, the dose-dependent reduction in lung coefficients observed in female rats suggests potential pulmonary sensitivity. However, the complete reversibility following a 4-week recovery period and the absence of histopathological abnormalities collectively indicate that these changes do not reflect structural damage.

After entering the body via the oral route, drug-induced toxicities may arise either from the parent form or its biotransformation derivatives [24]. Most pharmaceuticals undergo Phase I oxidative metabolism, generating reactive intermediates such as quinones and epoxides that mediate tissue damage via covalent adduction to cellular macromolecules or the induction of oxidative cascades [25]. Notably, the Phase II metabolic enzymes can mediate the binding reactions of endogenous substances or exogenous compounds, such as glucuronidation and sulfation, which convert the parent drug into polar, readily excretable metabolites, contributing to the excretion of the drug, which may partially explain the toxicity of drugs from one perspective [26,27]. The Phase I metabolites of rhein, including rheinhydroxylate and its tautomers, were reported to possess hepatotoxicity risk, while the toxicity risk induced by the Phase II metabolites, such as rheinglucoside, rheinglucuronic acid, and rhein sulfate, is small [28,29,30]. Therefore, to a certain extent, investigating the metabolic processes of 221s (2,9) may provide further mechanistic insights into their potential toxicological implication. Currently, research methods for drug metabolism primarily encompass in vivo and in vitro metabolism studies [31,32]. In vivo metabolism research can reflect the characteristics of drug metabolism effectively, comprehensively, and accurately [33]. Thus, this work investigates the metabolites of 221s (2,9) following gavage administration to rats.

UPLC-Q-TOF-MS technology was employed to analyze the metabolites of 221s (2,9) in plasma, urine, bile, and feces samples collected from SD rats. The metabolic pathways of 221s (2,9) in rats are depicted in Figure 6. A total of 31 metabolites were identified, with 16, 9, 19, and 16 metabolites detected in blood, urine, bile, and feces, respectively. It was found that the primary metabolic reactions of 221s (2,9) include oxidation, hydrolysis, methylation, glucuronidation, sulfation, and cysteine conjugation. Compound 221s (2,9) readily forms conjugated metabolites, such as methylated products, sulfated products, and glucuronidated products, which is consistent with the metabolic pathways of danshensu reported [34].

The liver is the primary organ responsible for drug metabolism [35]. Bile, as a major excretion route for xenobiotics and their metabolites, plays a pivotal role in systemic clearance and potential enterohepatic recirculation [36]. The detection and characterization of metabolites in bile hold significant implications for understanding the hepatic processing and elimination mechanisms of 221s (2,9) in rats. Partial Phase I metabolites and most Phase II metabolites, especially glucuronic acid metabolites, can be detected in rat bile, which is consistent with the literature reports that bile excretion is the main route for hydrophilic conjugates [37]. This also suggests that the drug may be primarily cleared through bile excretion, potentially due to its larger molecular weight or higher lipophilicity.

The detection of glucuronide conjugates and sulfate conjugates of 221s (2,9) in fecal samples indicates that some of the metabolites excreted by bile escape enterohepatic recirculation and are ultimately excreted through feces. The presence of 221s (2,9) in feces suggests that the Phase II metabolites excreted in bile, such as glucuronide conjugates and sulfate conjugates, are partially hydrolyzed by intestinal bacterial β-glucuronidase or sulfatase [38], thereby releasing the parent compound.

Pharmacokinetic profiling revealed that 19 out of 31 identified metabolites were sulfated and glucuronidated conjugates, which exhibited markedly improved aqueous solubility and molecular polarity characteristics. This biochemical transformation significantly improves excretion kinetics, thereby reducing the risk of target organ accumulation and associated toxicodynamic liabilities for compound 221s (2,9) in body. Consequently, this biotransformation pattern may constitute a fundamental mechanism underlying the demonstrated low toxicity profile of 221s (2,9) in preclinical animal models.

Although 221s (2,9) demonstrated low toxicity and favorable safety characteristics in toxicological evaluations, with its metabolites predominantly consisting of Phase II metabolites, the analysis of metabolites alone is insufficient to establish a complete mechanistic framework of toxicity. Future research will transcend static metabolite identification to focus on the regulatory mechanisms governing the pharmacokinetic–toxicokinetic relationship of 221s (2,9), mediated through the dynamic metabolism–transport interplay. Through an in-depth investigation of this network, the molecular basis underlying the toxic effects of 221s (2,9) in vivo will be systematically revealed, thereby providing a more comprehensive scientific basis for its safety evaluation and preclinical research.

In summary, this study comprehensively evaluated the safety profile of 221s (2,9), a novel danshensu-derived antihypertensive agent. Acute toxicity studies in mice established a maximum tolerated dose (MTD) of 3000 mg/kg with no observed mortality or significant adverse effects. A 12-week chronic toxicity study in rats revealed that the compound induced only minor, often sex-specific, and generally reversible alterations in hematological and biochemical parameters, with no associated histopathological evidence of organ damage. Furthermore, metabolomic analysis identified 31 metabolites, with the majority being Phase II conjugates (e.g., sulfation, glucuronidation). The prominence of these hydrophilic, readily excretable metabolites is proposed as a key mechanistic explanation for the compound’s low toxicity, as it minimizes systemic accumulation and potential target organ damage. In conclusion, the integrated toxicological and metabolomic data strongly support the favorable preclinical safety profile of 221s (2,9), providing a solid foundation for its future clinical translation.

## 4. Conclusions

In this study, for the first time, the favorable safety profile of 221s (2,9) was confirmed through acute and long-term toxicity studies. The MTD was determined to be 3000 mg/kg in single-dose acute toxicity. Long-term toxicity investigations further demonstrated no significant alterations in general behavioral characteristics, spontaneous locomotor activity, or central nervous system function in experimental animals. Additionally, no toxicity-related changes were observed in serum biochemical parameters or histopathological examinations of major organs. Metabolic profiling revealed that Phase II metabolic pathways—primarily generating sulfate esters and glucuronide conjugates—serve as the dominant biotransformation routes, which may underlie the low toxicity manifestation of 221s (2,9). Future investigations will delineate the low toxicity mechanism through metabolism–transport interplay to support clinical translation assessment of this candidate compound.

## 5. Materials and Methods

### 5.1. Materials

#### 5.1.1. Chemical and Reagents

Compound 221s (2,9) (purity > 99.5%) was chemically synthesized and provided by Xi’an key laboratory of multi-synergistic antihypertensive innovative drug development of Xi’an Medical University (Xi’an, China). HPLC-grade acetonitrile and methanol were purchased from Thermo Fisher Scientific Co. (Beijing, China). All other chemicals were of HPLC grade.

#### 5.1.2. Animals

Adult-specific pathogen-free (SPF) Kunming mice (20 ± 2 g) and Sprague–Dawley (SD) rats (180–200 g) and mice were purchased from the Experimental Animal Center of Xi’an Jiaotong University (Xi’an, China) (permit number SCXK 2017–003) and housed under controlled conditions (25 °C, 50 ± 5% humidity, and 12 h dark–light cycles). The animal study protocol was approved by the Institutional Review Board of Xi’an Medical University (protocol code XYLS2022053) for studies involving animals.

### 5.2. Methods

#### 5.2.1. Synthesis of 221s (2,9)

**Scheme 1:**
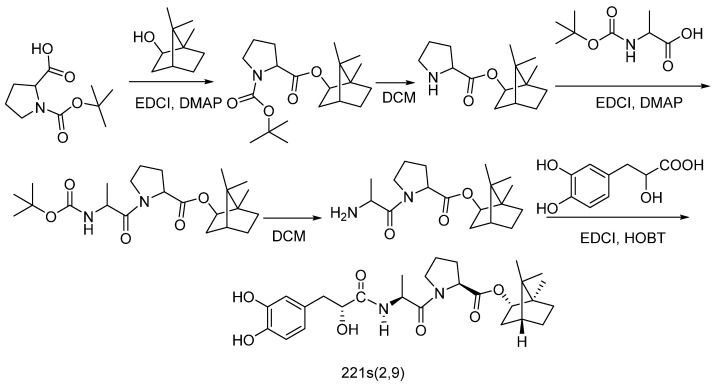
Synthesis of 221s (2,9) [14].

Compound 221s (2,9) was synthesized according to the literature [14] via a five-step sequence (Scheme 1), as follows: (1) Boc-proline coupling with borneol via EDCI/DMAP in DCM (83.6% yield); (2) deprotection with H3PO4 to yield intermediate 2 (99.6% yield); (3) alanine conjugation using EDCI/DMAP in DMF/DCM (79.0% yield); (4) acidic deprotection forming intermediate 4 (79.6% yield); and (5) final coupling with danshensu employing HOBT/EDCI in DMF under ice-bath conditions, affording 221s (2,9) as white solid after work-up (59.7% yield).

#### 5.2.2. Acute Toxicity

##### Determination of the Median Lethal Dose (LD_50_)

After acclimation for one week, 80 SPF-level KM mice (20 ± 2 g) were randomly assigned to a control group and three treatment groups (*n* = 20 per group) (10 males and 10 females in each group). For the treatment group, the mice were given 221s (2,9) (2000 mg/kg, 1320 mg/kg, and 871.2 mg/kg). Mortality and general health status were continuously monitored for 7 consecutive days.

##### Determination of Maximum Tolerated Dose (MTD)

After acclimation for one week, 40 SPF-level KM mice (20 ± 2 g) were randomly assigned to control and treatment groups (*n* = 20 per group, balanced by sex). Following a 12 h fasting period, the treatment group received a single intragastric dose of 3000 mg/kg. After treatment, the mice were continuously monitored at 0.5, 1, 2, 4, 6, 24, 48, 72, 96, 120, 144, and 168 h for mortality, intoxication symptoms, and behavioral parameters. Terminal necropsy was performed under isoflurane anesthesia. The major organs (heart, liver, kidney, lung) were excised and weighed for organ-to-body-mass index calculation. The tissues were fixed and stained with hematoxylin-eosin (HE) for histopathological evaluation.

#### 5.2.3. Long-Term Toxicity

##### Animal Grouping and Dosing

After acclimation for one week, a total of 160 Sprague–Dawley rats (80 males and 80 females) were randomly assigned to four experimental groups (*n* = 40 per group, balanced for sex): (1) CNTR group, given saline for 12 weeks; (2) 221s (2,9)-H group, given high-dose oral administration of 221s (2,9) 80 mg/kg for 12 weeks; (3) 221s (2,9)-M group, given medium-dose oral administration of 221s (2,9) 40 mg/kg for 12 weeks; and (4) 221s (2,9)-L group, given low-dose oral administration of 221s (2,9) 20 mg/kg for 12 weeks. Following the treatment phase, a 4-week recovery period was implemented to observe the potential reversibility of treatment-related effects, during which all animals continued to be monitored under identical housing conditions without any intervention.

##### Clinical Observation

At the end of treatment, half of the rats in each group were euthanized via CO_2_. At the end of the recovery period, the remaining half of the rats were euthanized via CO_2_. All blood samples were collected and centrifuged for the detection of hematological parameters, including the white blood cell (WBC) counts, the red blood cell (RBC) counts, mean corpuscular hemoglobin (MCH), mean corpuscular volume (MCV), and so on, which were determined using an automated hematology analyzer (BC-500, Mindray, China). The biochemical parameters, including alanine transaminase (ALT), aspartate transaminase (AST), alkaline phosphatase (ALP), total bilirubin (TBIL), total protein (TP), total cholesterol (TC), triglycerides (TG), urea nitrogen (UREA), creatinine (CREA), glucose (Glu), albumin (Alb), potassium (K), and sodium (Na), were determined using an automated biochemistry analyzer (BS-330E, Mindray, Shenzhen, China).

The major organs (heart, liver, kidneys, lungs) were excised and weighed for organ-to-body-mass index calculation. The tissues were fixed and stained with hematoxylin-eosin (HE) for histopathological evaluation.

#### 5.2.4. The In Vivo Metabolic Pathways of 221s (2,9)

##### Sample Collection

After acclimation for one week, 20 rats were fasted overnight and randomized into a treatment group and a control group. For the treatment group, 15 rats (5 rats for blood samples, 5 rats for urine and feces samples, and 5 for bile samples) were given 221s (2,9) (30 mg/kg) orally, which was prepared as previously described, while the control group rats were administered saline in the same manner.

The blood samples were collected in heparinized tubes pretreated with NaF at 0, 0.5, 1, 2, 4, 6, 12, and 24 h after administration. After centrifugation at 3500 rpm for 10 min, the supernatant plasma was obtained and stored at −80 °C for further use. For urine and feces samples, individual rats were housed in metabolic cages to ensure accurate and contamination-free sample collection. Prior to oral administration with 30 mg/kg 221s (2,9), the rats were fasted for 12 h and allowed to acclimate to the cages and to empty their bladders to minimize baseline urine interference. The urine and feces samples were collected during 0–6, 6–12, and 12–24 h after drug administration. For the collection of bile samples, the rats were anesthetized with isoflurane and subjected to surgical procedures to cannulate the common bile duct. Following adequate anesthesia, a midline abdominal incision was made to expose the bile duct and insert sterile polyethylene tubing into it. The free end of the tubing was connected to a collection container placed outside of the cage. The bile duct samples were collected during 0–6, 6–12, and 12–24 h after drug administration. All collected samples were stored at −80 °C.

##### Sample Pretreatment

The fecal samples were weighed and homogenized with four times the volume of methanol by ultrasonication for 1 h to obtain fecal homogenates. All samples (plasma, urine, bile, and fecal homogenates) were then mixed with four times the volume of methanol and centrifuged at 14,000 rpm for 5 min to obtain plasma, urine, bile, and fecal samples. These samples were stored at −80 °C until further analysis.

##### Instruments and Analytical Conditions

The UPLC-MS/MS system consists of a Waters ACQUITY UPLC H-Class PLUS Instrument and an Xevo G3 QT of mass. A Waters BEH C18 (2.1 × 100 mm, 1.7 μm) maintained at 40 °C was applied for chromatographic separation. The mobile phase consisted of deionized water with 0.1% formic acid (solvent A) and acetonitrile (solvent B). Separation was performed at a flow rate of 0.4 mL/min and an injection volume of 5 μL with the following gradient elution: 0.0–2.0 min, 95% solvent A; 2.0–7.0 min, 95–5% solvent A; 7–9 min, 5–95% solvent A; and 9–10 min 95% solvent A.

Mass detection was carried out using positive ion electrospray ionization (ESI). The optimal parameters for MS were as follows: capillary voltage, 3000 V; source temperature, 120 °C; cone gas flow, 50 L/h; desolvation temperature, 450 °C; desolvation gas flow, 900 L/h.

Micromass MarkerLynx Application Version 4.1 was used to analyze Raw UPLC/MS data. MarkerLynx served as the tool for selecting and aligning peaks. The initial dataset underwent processing with the following specified parameters: a starting retention time of 0 min, a concluding retention time of 10 min, a mass tolerance set at 0.02 Da, and a retention time tolerance of 0.1 min. Subsequently, the raw data were transformed into a unified matrix, where aligned peaks sharing the same mass-to-retention-time pairs were included, along with their normalized intensities and sample identifiers. This resultant three-dimensional dataset underwent unsupervised Principal Component Analysis (PCA).

The ions associated with 221s (2,9) were pinpointed based on their high mass accuracy (less than 3 ppm) and PCA of UPLC/MS data derived from the urine samples collected over a 24 h period from rats administered either a vehicle or 30 mg 221s (2,9) per kg of body weight. The ions that significantly contributed to the grouping separation in the PCA loadings plot and those exclusively observed in the dosed samples were deemed as 221s (2,9) or its metabolites. The ions in the PCA loadings plot that most influenced the clustering were manually reviewed from the raw data to validate their authenticity as analytical peaks and to confirm their mass accuracy.

#### 5.2.5. Statistical and Data Analysis

Statistical analyses were conducted using GraphPad Prism (Version 9.5.0) and IBM SPSS Statistics (Version 25). Quantitative data are presented as mean ± standard deviation (SD). Differences among multiple groups were assessed using one-way ANOVA when variances were homogeneous. Otherwise, nonparametric tests were applied. For comparisons between two groups, the *t*-test was employed. A *p*-value of less than 0.05 was considered statistically significant, with the following significance thresholds: * *p* < 0.05, ** *p* < 0.01, *** *p* < 0.001, and **** *p* < 0.0001.

## Figures and Tables

**Table 1:** Effects of 221s (2,9) on hematological parameters in female rats.

Parameter	12 Weeks				16 Weeks			
	CNTR	221s (2,9)-H	221s (2,9)-M	221s (2,9)-L	CNTR	221s (2,9)-H	221s (2,9)-M	221s (2,9)-L
WBC/10^9^/L	18.98 ± 2.55	17.16 ± 4.27	11.74 ± 1.06 **	14.82 ± 2.33	16.45 ± 2.02	20.85 ± 5.20	11.46 ± 3.04	13.93 ± 1.31
Neu#/10^9^/L	4.09 ± 0.99	5.19 ± 2.21	2.44 ± 0.33 *	3.97 ± 2.63	3.73 ± 0.83	4.41 ± 0.98	2.87 ± 0.66	3.78 ± 0.38
Lym#/10^9^/L	14.32 ± 2.03	11.28 ± 4.00	8.65 ± 1.20 **	10.05 ± 2.37	12.00 ± 1.86	15.48 ± 4.29	7.75 ± 2.49	9.16 ± 1.31
Mon#/10^9^/L	0.54 ± 0.22	0.64 ± 0.32	0.61 ± 0.09	0.77 ± 0.14	0.67 ± 0.16	0.87 ± 0.26	0.80 ± 0.22	0.93 ± 0.05
Eos#/10^9^/L	0.02 ± 0.02	0.02 ± 0.01	0.02 ± 0.01	0.01 ± 0.01	0.02 ± 0.01	0.03 ± 0.02	0.02 ± 0.01	0.04 ± 0.03
Bas#/10^9^/L	0.01 ± 0.00	0.03 ± 0.01	0.02 ± 0.01	0.02 ± 0.02	0.03 ± 0.01	0.06 ± 0.02	0.03 ± 0.01	0.03 ± 0.00
Neu/%	21.62 ± 4.34	31.15 ± 15.51	21.10 ± 4.49	25.97 ± 14.37	22.8 ± 4.83	21.8 ± 3.54	25.5 ± 4.92	27.4 ± 3.64
Lym/%	75.47 ± 3.43	65.02 ± 15.16	73.40 ± 4.22	68.67 ± 14.24	72.78 ± 5.38	73.55 ± 3.38	67.08 ± 5.66	65.50 ± 3.93
Mon/%	2.83 ± 0.99	3.63 ± 1.34	5.22 ± 0.44 **	5.20 ± 0.76 **	4.15 ± 1.38	4.33 ± 1.09	6.98 ± 0.84	6.68 ± 0.70
Eos/%	0.08 ± 0.07	0.07 ± 0.05	0.14 ± 0.10	0.05 ± 0.05	0.13 ± 0.04	0.10 ± 0.07	0.15 ± 0.05	0.20 ± 0.19
Bas/%	-	0.13 ± 0.07	0.14 ± 0.10	0.12 ± 0.09	0.15 ± 0.05	0.23 ± 0.04	0.30 ± 0.10	0.23 ± 0.04
RBC/10^12^/L	6.72 ± 0.51	7.22 ± 1.15	6.94 ± 0.81	7.27 ± 1.12	6.51 ± 0.37	6.60 ± 0.32	6.17 ± 0.32	6.90 ± 0.29
HGB/g/L	145.33 ± 10.39	153.33 ± 26.12	157.60 ± 17.62	160.83 ± 19.83	140.25 ± 6.46	145.00 ± 6.67	135.25 ± 7.05	149.50 ± 2.06
HCT/%	31.63 ± 2.12	32.75 ± 5.11	33.58 ± 3.65	34.30 ± 4.39	30.75 ± 0.95	31.43 ± 1.35	29.70 ± 1.80	32.60 ± 0.52
MCV/fL	47.12 ± 0.99	45.38 ± 0.38	48.48 ± 0.55	47.33 ± 1.99	47.45 ± 1.55	47.65 ± 0.73	48.15 ± 1.17	47.33 ± 1.65
MCH/pg	21.63 ± 0.33	21.18 ± 0.24	22.74 ± 0.24	22.20 ± 0.92	21.55 ± 0.41	21.98 ± 0.51	21.90 ± 0.63	21.73 ± 0.69
MCHC/g/L	459.50 ± 3.95	466.83 ± 7.27	469.60 ± 3.26 *	469.00 ± 5.51 *	454.75 ± 6.98	461.00 ± 4.53	455.25 ± 7.12	458.75 ± 5.40
RDW-CV/%	12.47 ± 0.45	12.07 ± 0.31	11.84 ± 0.49	12.25 ± 0.42	11.50 ± 0.29	11.83 ± 0.24	11.55 ± 0.32	11.98 ± 0.15
RDW-SD/fL	26.52 ± 1.21	24.82 ± 0.90	25.74 ± 1.29	25.90 ± 0.87	24.70 ± 0.80	25.60 ± 0.68	25.33 ± 0.22	25.73 ± 0.87
PLT/10^9^/L	700.33 ± 81.90	659.50 ± 73.42	586.80 ± 123.15	614.83 ± 140.98	810.00 ± 40.07	718.75 ± 41.05	791.5 ± 92.04	798.5 ± 110.26
MPV/fL	6.68 ± 0.29	6.30 ± 0.46	6.64 ± 0.27	6.52 ± 0.16	6.40 ± 0.20	6.65 ± 0.11	6.58 ± 0.13	6.33 ± 0.16
PDW	15.32 ± 0.13	15.25 ± 0.13	15.26 ± 0.08	15.35 ± 0.30	15.18 ± 0.08	15.25 ± 0.11	15.23 ± 0.11	15.35 ± 0.11
PCT/%	0.47 ± 0.05	0.42 ± 0.06	0.39 ± 0.07	0.40 ± 0.10	0.52 ± 0.02	0.48 ± 0.04	0.52 ± 0.05	0.50 ± 0.06

Female rats were administrated with 221s (2,9) for 12 weeks, followed by a 4-week recovery period. * *p* < 0.05, ** *p* < 0.01 compared to CNTR.

**Table 2:** Effects of 221s (2,9) on hematological parameters in male rats.

Parameter	12 Weeks				16 Weeks			
	CNTR	221s (2,9)-H	221s (2,9)-M	221s (2,9)-L	CNTR	221s (2,9)-H	221s (2,9)-M	221s (2,9)-L
WBC/10^9^/L	14.36 ± 2.93	13.92 ± 2.10	15.70 ± 3.07	15.50 ± 3.73	17.01 ± 1.13	21.45 ± 5.82	14.50 ± 1.52	15.55 ± 1.22
Neu#/10^9^/L	2.52 ± 0.44	3.03 ± 0.56	2.75 ± 0.45	2.64 ± 0.38	3.37 ± 0.83	3.50 ± 0.61	2.76 ± 0.24	3.40 ± 0.36
Lym#/10^9^/L	11.19 ± 2.47	10.42 ± 2.37	12.20 ± 2.74	12.25 ± 3.71	12.63 ± 0.86	16.85 ± 5.12	10.76 ± 1.47	10.73 ± 1.52
Mon#/10^9^/L	0.64 ± 0.27	0.45 ± 0.18	0.72 ± 0.12	0.58 ± 0.19	0.94 ± 0.06	1.02 ± 0.18	0.94 ± 0.14	1.33 ± 0.15
Eos#/10^9^/L	0.01 ± 0.01	0.01 ± 0.01	0.01 ± 0.01	0.02 ± 0.01	0.02 ± 0.01	0.03 ± 0.01	0.01 ± 0.004	0.05 ± 0.03
Bas#/10^9^/L	0.01 ± 0.01	0.01 ± 0.00	0.02 ± 0.01	0.02 ± 0.01	0.05 ± 0.02	0.06 ± 0.02	0.03 ± 0.01	0.04 ± 0.01
Neu/%	17.83 ± 2.90	22.38 ± 5.69	17.88 ± 2.62	18.30 ± 5.53	19.73 ± 4.28	16.78 ± 2.04	19.28 ± 2.81	22.18 ± 3.94
Lym/%	77.70 ± 3.35	74.18 ± 6.84	77.35 ± 2.83	77.57 ± 6.52	74.38 ± 3.85	78.00 ± 2.21	73.98 ± 2.74	68.70 ± 4.94
Mon/%	4.40 ± 1.35	3.33 ± 1.49	4.67 ± 0.61	3.93 ± 1.67	5.53 ± 0.65	4.88 ± 1.01	6.45 ± 0.45	8.55 ± 0.95 *
Eos/%	0.03 ± 0.07	0.07 ± 0.07	0.03 ± 0.05	0.12 ± 0.07	0.13 ± 0.08	0.13 ± 0.04	0.08 ± 0.04	0.35 ± 0.26
Bas/%	0.03 ± 0.05	0.03 ± 0.05	0.07 ± 0.05	0.08 ± 0.04	0.25 ± 0.17	0.23 ± 0.04	0.23 ± 0.04	0.23 ± 0.08
RBC/10^12^/L	7.42 ± 0.71	7.31 ± 0.22	7.38 ± 0.49	7.10 ± 0.50	7.16 ± 0.43	7.06 ± 0.27	7.20 ± 0.15	6.75 ± 0.37
HGB/g/L	152.83 ± 13.90	153.17 ± 3.13	158.00 ± 12.81	150.00 ± 9.71	146.75 ± 9.91	141.75 ± 5.54	147.75 ± 2.49	140.75 ± 6.22
HCT/%	33.00 ± 2.69	32.82 ± 0.64	33.87 ± 2.73	32.02 ± 2.32	32.58 ± 2.13	31.73 ± 1.01	32.60 ± 0.45	30.65 ± 1.70
MCV/fL	44.50 ± 0.76	44.97 ± 0.91	45.82 ± 1.09	45.18 ± 1.88	45.55 ± 1.21	45.00 ± 0.94	45.25 ± 0.84	45.45 ± 0.48
MCH/pg	20.60 ± 0.24	20.98 ± 0.32	21.40 ± 0.52	21.13 ± 0.80	20.55 ± 0.54	20.08 ± 0.39	20.53 ± 0.30	20.85 ± 0.45
MCHC/g/L	462.83 ± 5.93	467.00 ± 5.89	466.83 ± 4.14	467.83 ± 6.89	451.75 ± 3.56	446.50 ± 6.22	454.00 ± 5.05	458.50 ± 9.12
RDW-CV/%	13.20 ± 0.37	13.52 ± 0.40	12.97 ± 0.49	13.97 ± 1.16	12.98 ± 0.29	13.38 ± 0.68	14.00 ± 0.25	13.98 ± 0.77
RDW-SD/fL	26.52 ± 0.77	27.32 ± 1.07	26.52 ± 0.74	28.02 ± 1.38	26.50 ± 1.10	27.25 ± 1.53	28.53 ± 0.15	28.55 ± 1.16
PLT/10^9^/L	716.67 ± 153.32	661.17 ± 68.05	661.67 ± 103.16	708.17 ± 104.08	761.25 ± 78.06	781.50 ± 106.57	749.50 ± 71.09	817.75 ± 12.54
MPV/fL	6.70 ± 0.18	6.78 ± 0.22	6.60 ± 0.28	6.72 ± 0.34	6.75 ± 0.15	6.63 ± 0.13	6.55 ± 0.18	6.65 ± 0.11
PDW	15.22 ± 0.18	15.15 ± 0.10	15.20 ± 0.13	15.10 ± 0.15	15.15 ± 0.05	15.20 ± 0.07	15.15 ± 0.05	15.13 ± 0.08
PCT/%	0.48 ± 0.10	0.45 ± 0.04	0.44 ± 0.08	0.47 ± 0.08	0.51 ± 0.06	0.52 ± 0.08	0.49 ± 0.03	0.54 ± 0.01

Male rats were administrated with 221s (2,9) for 12 weeks, followed by a 4-week recovery period. * *p* < 0.05 compared to CNTR.

**Table 3:** UPLC-MS data of 221s (2,9) metabolites in rats.

Peak	t_R_/min	Formula	Theoretical Mass *m*/*z*	Experimental Mass *m*/*z*	Error (ppm)	MS/MS Fragment Ions	Metabolites Detected in Matrices
M0	7.09	C_27_H_39_N_2_O_7_^+^	503.2757	503.2758	0.2	367.1498, 252.1953, 224.0921, 137.1316, 116.0703	Plasma, Feces
M1	3.73	C_10_H_19_O_4_S^+^	235.1004	235.0992	−5.1	155.1459, 137.1304, 81.0689	Plasma, Bile, Urine, Feces
M2	5.60	C_16_H_27_O_7_^+^	331.1756	331.1748	−2.4	155.1463, 137.1306, 81.0713	Plasma, Bile, Feces
M3	3.55	C_16_H_27_O_8_^+^	347.1706	347.1713	2.0	171.1455, 155.1461, 137.1305, 81.0699	Bile, Feces
M4	3.74	C_16_H_27_O_8_^+^	347.1706	347.1712	1.7	171.1452, 155.1461, 137.1307, 81.0642	Plasma, Urine, Feces
M5	4.01	C_16_H_27_O_8_^+^	347.1706	347.1708	0.6	171.1452, 155.1456, 137.1306, 81.0708	Urine, Feces
M6	4.35	C_16_H_27_O_8_^+^	347.1706	347.1707	0.3	171.1455, 155.1466, 137.1305, 81.0621	Bile, Feces
M7	4.61	C_16_H_27_O_8_^+^	347.1706	347.1694	−3.5	171.1458, 155.1467, 137.1300, 81.0714	Plasma, Bile, Feces
M8	4.91	C_16_H_27_O_8_^+^	347.1706	347.1728	6.3	171.1454, 155.1463, 137.1308, 81.0702	Urine, Feces
M9	3.46	C_17_H_23_N_2_O_7_^+^	367.1505	367.1493	−3.3	252.0880, 224.0908, 116.0696, 70.0638	Plasma, Feces
M10	3.88	C_18_H_25_N_2_O_7_^+^	381.1661	381.1628	−8.7	266.0998, 238.1053, 116.0699, 70.0640	Urine, Bile
M11	3.11	C_18_H_25_N_2_O_8_^+^	397.1610	397.1597	−3.3	282.0818, 254.0692, 116.0537, 70.0637	Urine
M12	3.48	C_17_H_23_N_2_O_10_S^+^	447.1073	447.1069	−0.9	367.1480, 332.0825, 252.0854, 116.0627, 70.0653	Bile
M13	3.55	C_18_H_25_N_2_O_10_S^+^	461.1229	461.1256	5.9	381.1643, 318.0637, 238.1075, 116.0693, 70.0635	Plasma
M14	3.90	C_18_H_25_N_2_O_10_S^+^	461.1229	461.1245	3.5	381.1636, 318.0641, 238.1072, 116.0689, 70.0635	Plasma
M15	3.09	C_20_H_28_N_3_O_9_S^+^	486.1546	486.1545	−0.2	371.0909, 343.0982, 116.0703,70.0637,	Bile
M16	7.44	C_28_H_41_N_2_O_7_^+^	517.2913	517.2914	0.2	381.1663, 266.1040, 252.1957, 238.1068, 116.0700, 81.0697	Plasma, Bile
M17	5.05	C_28_H_41_N_2_O_8_^+^	533.2862	533.2901	7.3	492.1459, 422.8778, 381.1678, 268.1917, 116.0706	Plasma
M18	5.65	C_28_H_41_N_2_O_8_^+^	533.2862	533.2884	4.1	492.1465, 422.8724, 381.1683, 268.1912, 116.0700	Plasma
M19	7.02	C_28_H_41_N_2_O_8_^+^	533.2862	533.2888	4.9	397.1460, 252.1971, 116.0703.	Plasma, Bile
M20	7.10	C_28_H_41_N_2_O_8_^+^	533.2862	533.2895	6.2	397.1464, 252.1958, 116.0705	Bile, Feces
M21	2.95	C_23_H_31_N_2_O_13_^+^	543.1825	543.1838	2.4	367.1484, 252.0841, 224.0886, 116.0688, 70.0620	Bile
M22	3.33	C_23_H_31_N_2_O_13_^+^	543.1825	543.1807	−3.3	367.1496, 252.0869, 224.0896, 116.0689, 70.0631	Bile
M23	4.15	C_23_H_31_N_2_O_13_^+^	543.1825	543.1815	−1.8	367.1489, 252.0857, 224.0900, 116.0693, 70.0635	Bile, Plasma
M24	3.38	C_24_H_33_N_2_O_13_^+^	557.1982	557.1971	−2.0	442.1329, 381.1643, 266.1020, 238.1037, 116.0690	Bile, Plasma
M25	3.60	C_24_H_33_N_2_O_13_^+^	557.1982	557.1973	−1.6	442.1301, 381.1646, 266.1003, 238.1060, 116.0694	Bile, Plasma
M26	6.10	C_30_H_44_N_3_O_9_S^+^	622.2798	622.2793	−0.8	486.1555, 371.0884, 325.0864, 252.0950, 116.0697, 81.0687	Feces, Bile
M27	6.22	C_30_H_44_N_3_O_9_S^+^	622.2798	622.2813	2.4	486.1303, 371.0802, 343.1038, 252.0927,116.0680	Urine, Feces
M28	6.56	C_33_H_47_N_2_O_13_^+^	679.3077	679.3076	−0.1	503.2747, 428.1180, 367.1491, 252.0944, 224.0898, 116.0694, 81.0683	Urine, Feces
M29	6.63	C_34_H_49_N_2_O_13_^+^	693.3234	693.3259	3.6	517.2813, 442.1249, 381.1685, 266.1019, 252.0875, 116.0690, 81.0678	Feces
M30	6.02	C_37_H_54_N_5_O_13_S^+^	808.3438	808.3371	−8.3	672.3594, 557.1544, 252.1939, 81.0700	Plasma, Urine, Feces, Bile
M31	6.19	C_39_H_55_N_2_O_19_^+^	855.3398	855.3372	−3.0	679.3068, 543.1871, 503.2729, 428.1123, 367.1534, 252.1949, 116.0699	Bile, Feces

## Data Availability

The authors confirm that the data supporting the findings of this study are available within the article.

## Abbreviations

The following abbreviations are used in this manuscript:

LD_50_	Median Lethal Dose
MTD	Maximum tolerated dose
CREA	Creatinine
ALT	Alanine transaminase
AST	Aspartate transaminase
ALP	Alkaline phosphatase
TBIL	Total bilirubin
